# Correlation of Androgen Receptor Expression With Ki67 Proliferative Index and Other Clinicopathological Characteristics in Invasive Mammary Carcinomas

**DOI:** 10.7759/cureus.70867

**Published:** 2024-10-04

**Authors:** D Keerthana Devi, V Pavithra, Leena D Joseph, Chithra Bhanu Challa

**Affiliations:** 1 Department of Pathology, Sri Ramachandra Institute of Higher Education and Research, Chennai, IND

**Keywords:** androgen receptor, breast cancer, invasive mammary carcinoma, ki67 proliferative index, prognostic factors

## Abstract

Introduction

The clinical importance of androgen receptor (AR) status in breast cancer is uncertain. The existing knowledge regarding the association of androgen receptor expression with clinicopathological factors of breast cancer is also limited. The main aim of this study is to evaluate the AR expression in breast cancer and to correlate it with the Ki67 proliferative index and other clinicopathological prognostic factors.

Methods

The expression of AR was evaluated in 192 invasive mammary carcinoma cases and the expression patterns were correlated with various clinicopathological prognostic factors such as age, tumor size, pathological primary tumor (pT) stage, nodal status, histological grade, estrogen receptor (ER) expression, progesterone receptor (PgR) expression, human epidermal growth factor receptor 2 (Her2) status, molecular subtype, and Ki67 labeling index. Immunohistochemistry was performed to assess AR, ER, PgR, Her2, and Ki67 expression. The clinicopathological data required for the analysis were obtained from the laboratory information system.

Results

Out of the 192 breast carcinoma cases, 139 (72.4%) showed AR-positive expression. The average age of the AR-positive cases was slightly higher than the AR-negative cases. AR-positive tumors tended to have a lower histological grade and positive ER and PgR expression. The expression of AR did not correlate with tumor size, pT stage, nodal status, Her2 status, and Ki67 labeling index.

Conclusion

The expression of AR is noted in a significant proportion of breast carcinoma cases. AR expression may be related to good prognostic factors such as ER expression, PgR expression, and lower histologic grade. We also observed that AR expression did not have any association with the Ki67 proliferative index.

## Introduction

The global burden of carcinoma of the breast is rising due to an increase in the incidence rates in underdeveloped parts of the world. Carcinoma of the breast is the most frequent and deadly malignancy occurring in females globally [1]. According to global cancer statistics in the year 2022, 2.3 million women were diagnosed with breast cancer and 6,70,000 women died due to the disease [2]. Breast cancer is a heterogenous neoplasm, with varying clinicopathological characteristics and molecular profiles, displaying a differing response to therapy and having different outcomes. The treatment decision and prognosis depend on various factors such as histological type, grade, stage of the disease, the status of hormonal receptors, Her2 status, and the Ki67 labeling index [3].

The growth of breast cancer is dependent on steroid hormones. The steroid hormone receptor family consists of estrogen receptor (ER), progesterone receptor (PgR), and androgen receptor (AR) [4]. The essential role of ER and PgR in the growth of breast cancer is well acknowledged [5]. They regulate cell proliferation and differentiation in breast cancer. Hence, antiestrogen treatment has been used to treat patients with hormone receptor-positive breast cancer effectively [6]. The significance of ER and PgR in breast cancer as a prognostic and theranostic marker is well established, but the importance of androgen receptor (AR) is uncertain. A meta-analysis by Vera-Badillo et al. that collected data from 19 studies and 7,693 patients demonstrated that AR positivity was found in 60.5% of breast cancer cases [7]. Vera-Badillo et al. also stated that AR positivity was more commonly detected in ER-positive tumors compared to ER-negative tumors (74.8% vs 31.8%) [7].

Various studies suggest that females with increased androgen levels have an elevated risk of breast cancer [8,9]. Some studies state that AR signaling has an anti-tumor effect in estrogen-dependent cancers [10]. However, over-expression of AR in ER-positive breast cancer leads to increased resistance to antiestrogen therapy, and hence treatment with AR antagonists can overcome this resistance [11]. In ER-positive tumors, AR expression is correlated with the small size of the tumor (≤2cm), lower TNM (Tumor, Node, Metastasis) stage, lower histological grade, and better outcome [12]. The existing data on the prognostic significance of AR in Her2neu-amplified breast tumors are somewhat conflicting, as certain studies show no correlation with survival, whereas some studies show an association with poorer outcomes [11]. Androgens can induce growth of AR-positive/ ER-negative cancer cells and this effect can be inhibited by the use of antiandrogens [13]. In triple-negative breast cancers (TNBC), AR expression is correlated with a lower Ki67 labeling index, lower stage, lower histological grade, and favorable outcomes [14]. These above findings suggest that androgens play an important role in breast tumorigenesis and AR can serve as a therapeutic target as well as a prognostic marker in breast cancer [15].

Ki-67 is a nuclear protein expressed in all proliferative phases of the cell cycle, except the G0 stage. Ki67 expression is a reliable marker for accurately identifying the proportion of cells with proliferative capacity in a tumor [16]. Immunohistochemistry is used to evaluate the Ki67 expression as a labeling index in tissue samples [16]. According to studies done by Soliman et al. and Alco et al., a high Ki67 labeling index (≥15%) was associated with poorer prognostic factors such as higher histological grade, ER negativity, and increased incidence of recurrence [17,18]. In breast cancer, the Ki67 expression helps in predicting the prognosis and deciding the treatment plans [18].

The current study aimed to assess the proliferative behavior of AR-positive breast tumors by correlating AR expression with the Ki67 labeling index. In this study, AR expression was also correlated with various other clinicopathological prognostic factors such as age, tumor size, pT stage, nodal status, histological grade, ER expression, PgR expression, human epidermal growth factor receptor 2 (Her2) status, and molecular subtype.

## Materials and methods

The retrospective observational study was conducted in the Department of Pathology, Sri Ramachandra Medical College and Hospital, Chennai. This study was done on 192 mastectomy specimens of invasive mammary carcinoma received in the Pathology Department from January 2022 to January 2024. The 192 cases were selected according to the predetermined inclusion and exclusion criteria. 

*Inclusion criteria*: All mastectomy cases with histopathological diagnosis of invasive mammary carcinoma were included in this study.

*Exclusion criteria*: All inflammatory lesions, benign tumors, post-chemotherapy specimens, and metastatic breast cancer cases were excluded from the study. All core biopsy cases with invasive mammary carcinoma were also excluded from this study. 

Clinicopathological prognostic characteristics for all 192 cases such as age, tumor size, pT stage, lymph nodal involvement, and histological grade were collected from the hospital information system.

Immunohistochemistry staining and interpretation

We evaluated ER, PgR, Her2, AR, and Ki67 expression by performing immunohistochemical analysis. Formalin-fixed, paraffin-embedded tissue blocks of the 192 mastectomy specimens were used for the immunohistochemical study. Primary antibodies for ER (Rabbit Monoclonal, Clone SP1; Roche, Basel, Switzerland), PgR (Rabbit Monoclonal, Clone 1E2; Roche, Basel, Switzerland), Her2 (Rabbit Monoclonal, Clone 4B5; Roche, Basel, Switzerland), AR (Rabbit Monoclonal, Clone EP120, Path in situ), and Ki67 (Rabbit Monoclonal, Clone 30-9; Roche, Basel, Switzerland) were used. The immunohistochemical investigation for all the cases was done in multiple batches. The positive control for ER, PgR, Her2, AR, and Ki67 was run whenever a new batch of cases was tested. Stain quality was assessed by two pathologists. We repeated immunohistochemistry for cases with suboptimal staining quality. ER and PgR expressions were interpreted using the Allred score method [19]. Her2 expression was interpreted using the College of American Pathologists' (CAP) 2018 guidelines [20]. AR expression was considered positive if ≥10% of cancer cells exhibited nuclear staining [21]. Ki67 labeling index was assessed by calculating the percentage of nuclei with positive staining in the tumor cells. The Ki67 index was assessed in the hotspot area (the proliferative area of the tumor) at 400x magnification and at least 500 cells were counted for each case. Ki67 proliferative index ≥15% was taken as high and <15 % was taken as low [18]. All the 192 cases were molecularly classified, based on the expression of ER, PgR, Her2, and Ki67 into luminal A, luminal B, Her2-enriched, luminal Her2 PgR positive, luminal Her2 PgR negative, and triple negative. The AR expression was correlated with the following factors: age, tumor size, pT stage, nodal status, histologic grade, ER expression, PgR expression, Her2 status, molecular subtype, and Ki67 labeling index.

Statistical analysis

Data collected by two researchers were then verified by a third researcher and statistician. The data collected were entered into Microsoft Excel 2018 (Microsoft Corp., Redmond, WA) and analyzed using SPSS software, version 16 (SPSS Inc., Chicago, IL). Any case of missing data was completely excluded from the statistical analysis. Shapiro-Wilk test was used to find the normal distribution. Continuous variables that follow a normal distribution, like age, were expressed as the mean and standard deviation. Continuous variables that do not follow a normal distribution, like tumor size, were expressed as a median. Descriptions of categorical variables like androgen receptor expression, pT stage, nodal status, histologic grade, molecular subtypes, ER status, PgR status, and Her2 status were expressed as frequency and proportion. A student’s t-test was employed to compare the two groups of means. The Mann-Whitney U test was employed to compare differences in a continuous but not normally distributed variable between two independent groups. A chi-square test was employed to compare two categorical variables. The Ki67 labeling index was analyzed as a categorical variable (high vs. low Ki67) and its correlation with AR expression was assessed using the chi-square test. To further investigate the association between AR expression and the Ki67 labeling index on a continuous scale, we compared the median Ki67 values between AR-positive and AR-negative groups using the Mann-Whitney U test. All tests were two-tailed and a p-value of less than 0.05 with a 95% confidence interval was considered statistically significant. 

Ethical consideration

The ethical approval for this study was obtained from the institutional research ethics committee (CSP-MED/24/AUG/107/241). The patient’s confidentiality and anonymity were maintained throughout the study.

## Results

**Figure 1:**
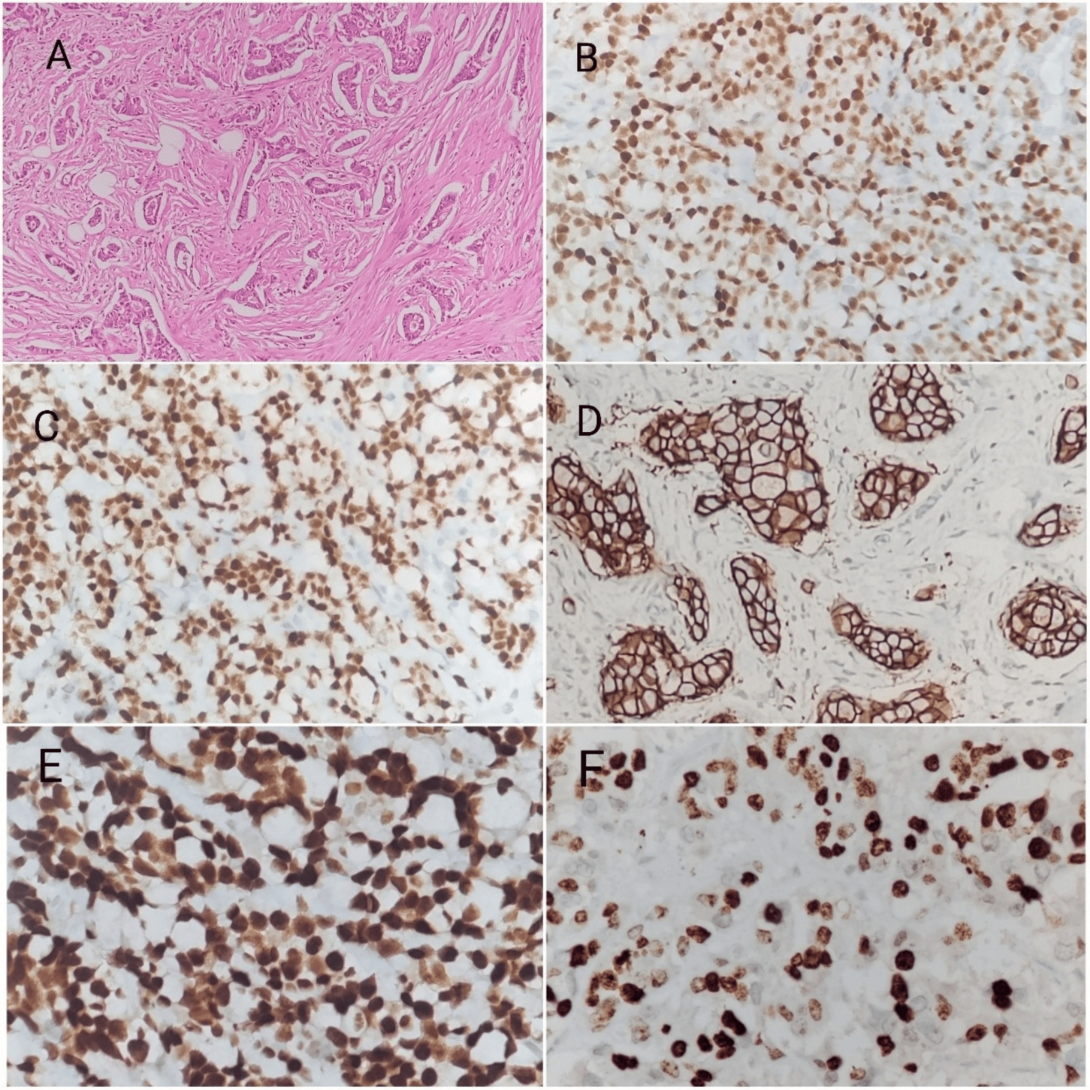
Histology of breast cancer and immunohistochemical expression of each marker in breast cancer (A) Histology of invasive ductal carcinoma (hematoxylin and eosin stain, 100 x magnification). (B) Immunohistochemical staining of ER showed nuclear expression, 100x magnification. (C) Immunohistochemical staining of PgR showed nuclear expression, 100x magnification. (D) Immunohistochemical staining of Her2 showed strong membranous expression, 200x magnification. (E) Immunohistochemical staining of AR showed nuclear expression, 400x magnification. (F) Immunohistochemical staining of Ki67 showed nuclear expression, 400x magnification. ER: estrogen receptor; PgR: progesterone receptor; Her2: human epidermal growth factor receptor 2; AR: androgen receptor.

In this study, 192 adult females with invasive mammary carcinoma were investigated. The average age of the study participants was 55.43 ± 11.43 years. Out of the 192 breast cancer patients, 139 (72.4%) cases were AR-positive and 53 (27.6%) cases were AR-negative. Figure 1 highlights the histology of breast cancer and immunohistochemical staining of ER, PgR, Her2, AR, and Ki67 in breast cancer.

Table 1 illustrates the correlation of AR expression with clinicopathological features of breast cancer. The mean age of the AR-positive cases was 56.78 ± 11.18 years, and the mean age of the AR-negative cases was 51.94 ± 11.43 years. The mean age was slightly higher among AR-positive cases when compared to AR-negative cases, and the difference is statistically significant with a p-value of 0.010. AR expression did not show any statistically significant correlation with tumor size (p-value:0.134). Among AR-positive tumors, 28 cases (20.1%) belonged to the pT1 stage and 86 cases (61.9%) belonged to the pT2 stage, whereas in AR-negative tumors, eight cases (15.1%) belonged to the pT1 stage and 31 cases (58.4%) to the pT2 stage. AR-positive tumors showed a slightly higher percentage of early-stage cases compared to AR-negative tumors. However, this finding did not have a statistically significant association (p=0.450). Of all AR-positive cases, 67 cases (48.2%) showed positive nodal status, and among AR-negative cases, 19 cases (35.8%) showed positive nodal status; however, this finding was not statistically significant with a p-value of more than 0.05. AR-negative cases had a higher percentage of grade 3 tumors than AR-positive cases (21/53 AR-negative cases (39.6%) vs. 21/139 AR-positive cases (15.1%)), and this finding was statistically significant with a p-value of less than 0.05. 

Positive AR expression was associated with ER and PgR expression with a significant p-value of less than 0.05. However, AR expression and Her2 status had no statistically significant correlation (p=0.465). The luminal B molecular subtype was the most common subtype among AR-positive tumors, which was found in 57 (41%) cases. In contrast, triple-negative was the common subtype in AR-negative tumors, which was seen in 27 (50.9%) cases. This finding was statistically significant (p=0.001). AR expression did not correlate with Ki67 low vs. high based on a 15% cut-off (p=0.703). However, to fully exclude correlation, we also compared median Ki67 values between AR-positive and AR-negative tumors. This additional analysis also showed no association between AR expression and the Ki67 labeling index (p=0.090). 

**Table 1:** Correlation of AR expression with clinicopathological parameters of invasive mammary carcinoma N: Total number of cases. Data is represented as n (frequency) and % (percentage); NA: Not applicable. AR: Androgen receptor; pT: pathological primary tumor; ER: estrogen receptor; PgR: progesterone receptor, Her2: human epidermal growth factor receptor 2.

Variables	Total (N=192)	n (%)		Z value	T value	Chi-square value	p-value
		AR-Positive (139 cases)	AR-Negative (53 cases)				
Mean age ± standard deviation	55.43 ± 11.43	56.78 ± 11.18	51.94 ± 11.43	NA	2.63	NA	0.010
Tumor size (cm) (median)	3.0	3.0	3.5	-1.49	NA	NA	0.134
pT staging							
pT1	36	28 (20.1%)	8 (15.1%)	NA	NA	2.64	0.450
pT2	117	86 (61.9%)	31 (58.4%)				
pT3	33	22 (15.8%)	11 (20.8%)				
pT4	6	3 (2.2%)	3 (5.7%)				
Nodal status							
Positive	86	67 (48.2%)	19 (35.8%)	NA	NA	2.37	0.124
Negative	106	72 (51.8%)	34 (64.2%)				
Histologic grade							
1	22	18 (13.0%)	4 (7.6%)	NA	NA	13.62	0.001
2	128	100 (71.9%)	28 (52.8%)				
3	42	21 (15.1%)	21 (39.6%)				
ER status							
Positive	121	103 (74.1%)	18 (33.9%)	NA	NA	26.53	<0.001
Negative	71	36 (25.9%)	35 (66.1%)				
PgR status							
Positive	112	97 (69.8%)	15 (28.3%)	NA	NA	27.16	<0.001
Negative	80	42 (30.2%)	38 (71.7%)				
Her2 status							
Positive	62	47 (33.8%)	15 (28.3%)	NA	NA	0.53	0.465
Negative	130	92 (66.2%)	38 (71.7%)				
Molecular subtype							
Luminal A	19	18 (13.0%)	1 (1.9%)	NA	NA	37.37	0.001
Luminal B	67	57 (41.0%)	10 (18.9%)				
Her2 enriched	27	19 (13.7%)	8 (15.1%)				
Luminal Her2 PgR positive	26	22 (15.8%)	4 (7.5%)				
Luminal Her2 PgR negative	9	6 (4.3%)	3 (5.7%)				
Triple-negative	44	17 (12.2%)	27 (50.9%)				
Ki67 labeling index							
<15%	33	23 (16.5%)	10 (18.9%)	NA	NA	0.14	0.703
≥15 %	159	116 (83.5%)	43 (81.1%)				
Ki67 labeling index (%) (median)	40	35	40	-1.69	NA	NA	0.090

## Discussion

The exact role of the androgen receptor and its ligand androgens in breast cancer pathogenesis remains controversial [4]. Androgens can influence breast cancer growth by a variety of mechanisms: by binding to AR and causing stimulation of neoplastic cell proliferation, by binding to ER and inhibiting the 17B-estradiol stimulatory effect on breast cancer cells, or by conversion to estradiol [22]. AR signaling also plays a crucial role in essential processes like DNA damage repair and cell cycle regulation in breast cancer [23]. Increased levels of serum androgens have been identified in women with metabolic syndrome. Metabolic syndrome has been recognized as an independent risk factor for breast cancer development [24]. Though AR is considered an emerging prognostic marker in breast cancer, there are conflicting published data regarding the correlation between AR expression and clinicopathological prognostic parameters of breast cancer patients. Certain previous studies have demonstrated AR expression with favorable outcomes in breast cancer [25,26].

This study analyzed AR expression and its association with various clinicopathological factors of breast carcinoma. A meta-analysis by Bozovic-Spasojevic et al. demonstrated that AR positivity was found in 58.6% of breast cancer cases [27]. According to a study done by Moinfar et al., 60% of all invasive carcinomas were found to be AR-positive [28]. In another study from India, done by Anand et al., the prevalence of AR expression was found to be 56% [29]. In our study, positive AR expression was found in 139/192 cases (72.4%) of invasive mammary carcinomas. Further, AR-positive expression was related to a slightly older age compared to AR-negative expression. This finding was contradictory with the studies done by Zhang et al. and Jia et al. in which the association of AR with age was statistically insignificant [30,31]. The AR-positive expression correlated with lower histological-grade tumors in our study. This finding was similar to another study done by Ogawa et al. where AR expression was correlated with low histologic grade [32]. In another study also, done by Agrawal et al., AR expression was associated with lower histological grade [4].

In our study, AR expression did not correlate with tumor size. In a study done by Zhang et al., there was no association between AR-positive expression and tumor size [30]. In our study, AR expression did not correlate with tumor stage (pT stage), which was in agreement with Anand et al.'s findings [29]. In our study, the AR expression was not related to lymph nodal involvement. This finding concurs with Zhang et al. [30]. However, Ogawa et al. reported that AR expression was related to negative nodal metastasis [32].

In our study, AR expression was related to ER and PgR positivity (favorable prognostic markers). This finding was in concordance with various other studies done by Park et al., Anand et al., and Ogawa et al. [21,29,32]. In the present study, AR expression did not correlate with Her2 status. This finding is similar to the results reported by Anand et al. and Ogawa et al [29,32]. However, our study found AR positivity in 19/27 cases (70.4%) of Her2 enriched, ER/PgR-negative breast cancers. Micello et al. reported that 77% of Her2-positive breast cancer cases showed AR expression [33], almost close to the results of our study. According to He et al., AR plays an essential role in promoting the proliferation of Her2-positive breast cancer [34]. These findings suggest that AR-targeted therapies may be used as an additional treatment for Her2-positive patients [34]. 

Triple-negative tumors are defined by the absence of expression of ER, PgR, and Her2. They account for about 20% of breast cancers. The only non-surgical treatment available for this subtype is platinum-based chemotherapy. Despite aggressive treatment, this subtype has a poor prognosis compared to other breast cancer subtypes [35]. Hence, it is essential to find additional biomarkers to predict the prognosis and drive molecular-targeted therapies in the triple-negative subset of patients. Of all the 192 breast carcinoma cases analyzed, 44 (22.9%) were categorized as belonging to the triple-negative subtype. Among the 44 triple-negative cases, 17 (38.6%) were AR-positive. The research by Luo et al. revealed that triple-negative breast cancer (TNBC) could be categorized into good and poor prognosis types based on AR expression [36]. Since AR positivity is noted in a significant number of TNBC cases, it may have a prognostic and therapeutic role in this subtype [32].

The cut-off point to differentiate high and low Ki67 expression in breast cancer is still a topic of controversy among researchers. According to the St. Gallen consensus in 2015, the majority of the experts agreed to a cut-off value of Ki67 within a range of 20%-29% [37]. However, several studies have revealed that the reproducibility of the Ki67 marker is low, especially in breast cancers with intermediate proliferation rates (15%-30%) [37,38]. According to International Ki67 Working Group (IKWG) and European Society for Medical Oncology (ESMO) guidelines, Ki67 labeling index of ≥30% is considered a high expression. However, the cut-off for low Ki67 expression according to IKWG is ≤5% and according to ESMO, it is ≤10% [39,40]. According to the Predict online decision tool for breast cancer, the Ki67 cut-off is 10% [41]. According to *Breast Tumors*, 5th edition, a part of the World Health Organization (WHO) Classification of Tumours series, a Ki67 cut-off of 14% or 15% has been suggested to distinguish between the luminal B subtype and the luminal A subtype [42]. 

In our study, the cut-off point ≥15% was used for Ki67 based on the findings of studies done by Soliman et al. and Alco et al. Soliman et al. reported that Ki67 of ≥15% was considered a high expression [18]. They also concluded that high Ki67 (≥15%) was associated with poor prognostic factors such as higher grade, ER/PgR negative expression, and increased incidence of metastasis and recurrence [18]. In another study done by Alco et al., four different cut-off points (≥10%, ≥15%, ≥20%, and ≥25%) for Ki67 were analyzed and the cut-off point of ≥15% was found to correlate with the highest number of adverse prognostic factors like younger age (≤40 years), histologic grade 3 tumors, ER negativity, and Her2 positivity [17].

In our study, AR expression and Ki67 labeling index did not have any statistically significant correlation. This finding was similar to the results obtained by Vera-Badillo et al. [43]. However, in a study done by Zhang et al., AR expression was associated with a lower Ki67 labeling index [30].

This study has certain limitations. Firstly, we could not correlate the AR expression with the overall survival and disease-free survival of the patients due to a lack of efficacy data. Secondly, since we had a significant difference in the total number of AR-positive and AR-negative cases, we could not comprehensively correlate AR expression with nodal status (AR expression with N0 vs N1 vs N2 vs N3 nodal status). Furthermore, we did not include metastatic patients in this study. Lastly, this study was a retrospective single institutional-based study with a modest sample size, which may restrict the generalizability of the results. Hence, a prospective multi-institutional study with a larger sample size is required to validate the prognostic significance of AR in breast cancer.

## Conclusions

AR expression is associated with favorable prognostic factors of breast cancer such as lower histologic grade, ER, and PgR expression. A significant proportion of Her2-positive and triple-negative breast cancer cases also show AR positivity. AR expression did not have any association with the Ki67 proliferative index. The immunohistochemical evaluation of AR status can help in predicting the prognosis as well as may lead to the use of new treatment modalities in breast cancer especially for triple-negative tumors. However, further studies are needed to find the exact role and efficacy of AR-targeted therapies in various molecular subtypes of breast cancer.
